# Newly Synthesized Water Soluble Cholinium-Purpurin Photosensitizers and Their Stabilized Gold Nanoparticles as Promising Anticancer Agents

**DOI:** 10.3390/ijms9050864

**Published:** 2008-05-23

**Authors:** Dorjnamjin Demberelnyamba, Mama Ariunaa, Young Key Shim

**Affiliations:** PDT Research Institute, School of Nano System Engineering, Inje University, Gimhae, South Korea

**Keywords:** Ionic liquid type photosensitizers, choline, purpurin-18, cholinium-purpurin-18, gold nanoparticles, anticancer agents

## Abstract

For possible future use in Photodynamic Therapy (PDT) and/or Photothermal Therapy (PTT) of cancer and screening of cancer cells a new type of ionic liquid photosensitizer –Cholinium-Purpurin-18 (Chol-Pu-18) – was synthesized and small gold (Au) nanoparticles, stabilized by this photosensitizer were prepared without adding any particular reducing agents and CTAB. UV-Vis spectroscopy and Transmission Electron Microscopy (TEM) were used for characterization of the nanoparticles and FAB-MS and NMR of the ionic liquid choline hydroxide, purpurin carboxylate and their ionic liquid type of photosensitizer were obtained.

## 1. Introduction

Noble metal nanoparticles exhibit a strong optical extinction at visible and near-infrared (NIR) wavelengths due to a localized surface plasmon resonance (LSPR) of their free electrons upon excitation by an electromagnetic field. Recent advances in the synthesis of noble metal nanoparticles have led to widespread interest in their properties and applications, as thoroughly reviewed by Daniel and Astruc [1].

Significant biomedical applications become possible when gold nanoparticle resonances are tuned to the near-infrared (NIR), where tissue is relatively transparent and gives better tissue transmission [2].

Photothermal therapies may benefit from the small size of gold nanoparticles in terms of their ability to permeate tissue and the leaky vasculature of tumors. Tunable gold nanoparticles could therefore serve as both a diagnostic and therapeutic technology for cancer treatment [3].

Apart from these inherent properties, a practical advantage of gold nanorods is that simple procedures which use cetyltrimethylammonium bromide (CTAB) surfactant as a soft template have been recently developed for high-yield nanorod synthesis [4]. However, biological applications have not been widely pursued with gold nanorods in part because nanorod solutions are stabilized by the presence of concentrated CTAB surfactant [5]. Although conjugates have been formed in the presence of CTAB [6], surfactant solutions are cytotoxic [7] and may interfere with established protein-linking protocols. A recent report explored the cytotoxicity of CTAB stabilized spherical gold nanoparticles [8].

Ionic liquids (ILs) are defined generally as salts composed of discrete cations and anions with melting points below 100°C, and many of them are liquids in room temperature [9]. Biologically active cations and anions have been used to make new ionic liquids. However, the primary driver for these materials has been the use of ions of known low toxicity to obtain the IL physical property set [10]. ILs with antimicrobial quaternary ammonium cations have been known and recently these have been shown to retain their biological activity [11].

When metal nanoparticles are prepared by chemical methods, the metal ions reduced by the boron containing reducing agents such as NaBH_4_ the resulting metal nanoparticles are contaminated with borides.

This reasons allows for the preparation of boride free metal nanopowders specially for use in biological and medical purposes.

We have therefore synthesized gold nanoparticles in aqueous system by using the newly synthesized water soluble ionic liquid-chlorin types of photosensitizer based on purpurin-18 from green algae *Spirulina maxima* and choline hydroxyde without adding any particular reducing agents and CTAB. Photodynamic therapy (PDT) treatment of cancer requires the avaibility of photosensitizers with high efficiency and selectivity for the destruction of tumor cells. Chlorins from Chlorophyll a are favourable photosensitizers, because they have a high extinction in the red light region, where light transmission through the human tissues is high. However, the chlorines and their derivatives have a serious problem in that they not soluble in water, so we tried to prepare water soluble chlorin derivatives by formation with IL based on the choline hydroxyde and chlorin derivatives such as Purpurin-18 (Pu-18).

These IL-purpurin derivatives simultaneously act both as the reductant and protective agent. Consequently, an external reductant is no longer needed, which significantly simplifies the process of pure nanoparticle preparation. A new Chol-Pu-18 having quaternary ammonium cations was synthesized and small gold (Au) nanoparticles prepared, stabilized by these IL-purpurin derivatives. UV-Vis spectroscopy and Transmission Electron Microscopy (TEM) were used for characterization of nanoparticles and NMR of IL, Pu-18 and IL-Purpurin derivatives.

## 2. Results and Discussion

**Scheme 1.**
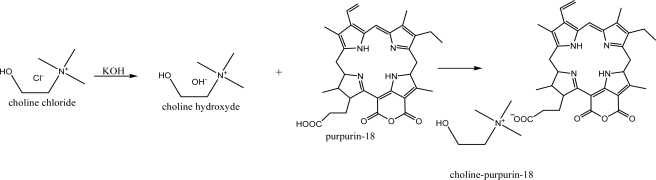
Pathways for the synthesis of cholinium–purpurin-18-carboxylate(Chol-Pu-18).

We have synthesized a new ionic liquid type photosensitizer cholinium-Purpurin-18 (Chol-Pu-18) as shown in Scheme 1.

The resulting Chol-Pu-18 compound had an extremely low chromatographic mobility, which confirmed the existence of a charge in the molecule. The NMR spectra of Chol-Pu-18 contains in addition to a Purpurin-18 structure an additional hydroxyl group and the singlet and triplets of the methyl and methylene groups at the nitrogen atom of the quaternary ammonium decisively confirmed the structure of the quaternary ammonium ionic liquid salt. The level of saturation of the porphyrin macrocycle strongly affects the overall nature of the absorption spectrum. Chlorins (2,3-dihydroporphyrins) are distinguished from the parent porphyrins by the presence of one reduced peripheral double bond, and this change in symmetry leads to a strong absorption in the long-wavelength portion of the visible spectrum (λ max = 650–680 nm) and particularly a number of new purpurin-18 derivatives showed their potential as second-generation photosensitizers (PS) for PDT.

**Figure 1.**
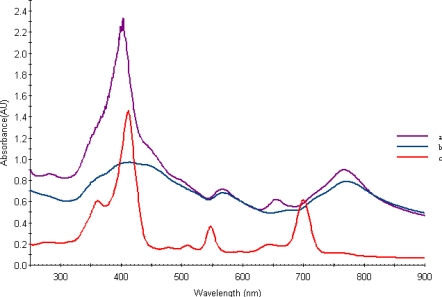
UV-Vis spectrum of ionic liquid type of Chol-Pur-18 photosensitizer (a), typical surface plasmon absorption spectrum of gold nanoparticles stabilized by Chol-Pur-18 photosensitizer (b) and Purpurin-18 (c) respectively.

In order to achieve a further bathochromic shift by ionic structure, the hydrogen of carboxylic group in purpurin-18 (max 700 nm) was replaced with a cholinium cation substituent. Figure 1b shows the UV-Vis absorption spectra obtained from aqueous Chol-Pur-18 photosensitizer solutions.

Thus, compared to Purpurin -18, these spectra of the Chol-Pu-18-carboxylate is broadened and gave a greater red shift of 67 nm. Compare with Purpurin-18 the Chol-Pu-18-carboxylate was found a red shifts from 547 to around 566 nm, from 644 nm to 654 nm and from 700 nm to 767 nm. These absorptions are obviously responsible for their red and green color respectively.

Compared to most of the chlorins, the ionic liquid type of purpurin photosensitizers showed a significant red shift of the long-wavelength absorption (Q-band) in their UV–Vis spectra, which makes them attractive candidates as PS for PDT.

Owing to their long wavelength absorptions in the red region, these compounds may have definite advantages over Photofrin® (a porphyrin derivative) for treating tumors which are deeply seated.

In conclusion, the new class of chlorins discussed here are the first examples of chlorins with cholinium IL structure.

This methodology has great potential in designing long wavelength absorbing and water soluble PS for PDT, and is currently being explored in our laboratory and biological PDT experiments with these compounds are currently in progress.

**Figure 2.**
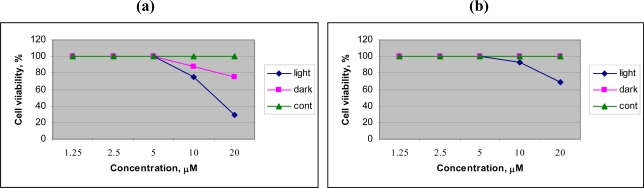
Viability of A549 cells treated with Chol-Pur-18 **(a)** and Chol-Pheophorbide a **(b)**. Curves are A549 cells after incubation with photosensitizers without illumination (dark), followed by illumination for 15 min (light) and control (cont) respectively

To evaluate the preliminary anticancer activity of Chol-Pur-18, and Cholinium salt of starting material Pheophorbide research was performed on human lung carcinoma cell, A596. The preliminary PDT research (Figure 2) of Chol-Pur-18 photosensitizer on A549 (human lung carcinoma cell) cells shows that the cytotoxic effects of Chol-Pur-18 were increased depending upon to their concentrations. The Chol-Pur-18 combines a moderate high cytophototoxicity (cell viability was 30% after 24 h) and low dark toxicity (cell viability was about 75%), compare with low cytophototoxicity cholinium salt of starting material of pheophorbide a (cell viability was 70%) in acute *in vitro* cell experiment. The Chol-Pur-18 possesses an intensive absorption band in the near infrared part of the spectrum (700 nm-800 nm) where biological tissues are transparent to a larger extend than in case of the first and second generation of PS (around 630 nm, 660–700 nm).

The reactions discussed above might also be useful for the preparation of other biological active compounds of natural and synthetic origins with different structure systems. Compared to insoluble in water chlorins and purpurins, we found that the IL form of the photosensitizers are water soluble and beneficial for future preparation of gold and other nanoparticles.

**Figure 3.**
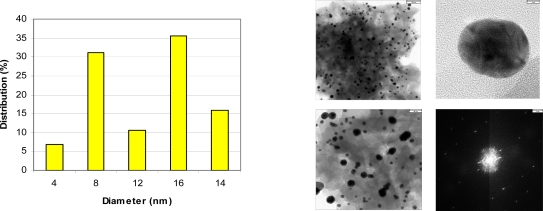
Representative size distribution histogram, TEM and Electron diffraction images of Au nanoparticles stabilized by Chol-Pur-18.

Figure 3 shows representative TEM, electron diffraction images and histogram of Chol-Pu-18 coated gold nanoparticles. The gold nanoparticles as shown in histogram are average size of 8–25 nm with reasonable narrow size distributions. The images can be seen that the gold nanoparticles are well separated, with no apparent sign of aggregation. Remarkably, the decahedral (pentagonal bipyramid) geometry is consistently the dominating morphology and widely observed in these gold nanoparticles and giving rise to additional plasmon bands and both the polar and the azimuthal dipolar plasmon bands red-shifted. These shifts in the peak plasmon absorbtion are related to a change in the local dielectric constant around the gold nanoparticles as a results of adsorption of Chol-Pu-18 electrolyte onto the gold nanoparticles. This was indeed observed in the UV-Vis-NIR spectra of gold nanoparticles in Figure 1b. The optical properties of gold decahedra shows that their sensitivity toward changes in the refractive index of the surrounding medium [13].

**Scheme 2.**
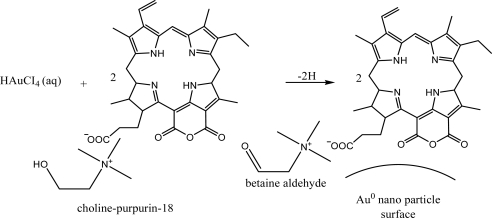
Cation coordination and Au nanoparticles stabilization mode

The functional groups are coordinated on the Au nanoparticles surfaces posses reverse micelle like structure (Scheme 2).

After the self reduction of the gold ions by hydroxyl group of cholinium-purpurin carboxylate stabilizers the surface of plasmon resonance band (λ_max_) around 520 nm broadened and little moved to the long wavelength region that indicating the formation of gold nanoparticles. The Soret band of porphyrin rings at 420 nm on the gold surfaces was broadened, indicating the formation of stacking structure of chlorin ring.

This pathway produced pure gold nanoparticles solutions with broad absorption maximum around at 750–800 nm.

Figure 1b shows the UV-Vis spectra of the Au nanopartcles stabilized by Chol-Pur-18 PSs. The surface plasmon absorption of gold nanoparticles have two bands: the strong long wavelength band in the near infrared region around 750–800 nm is due to the longitudinal oscillation of electrons and the short wavelength band in the visible region around 550 nm is due to the transverse electronic oscillation.

The prepared gold nanoparticles stabilized by IL-Chlorin particularly IL-Purpurin-18 photosensitizers may have dual effects as photosensitizer, photothermal destructor and may also use as contrast agents.

Many ionic liquid and ionic surfactant type of structure are also present in natural and synthetic compound molecules. Only problem has been asked of the possible toxicology of ionic liquid and ionic surfactant (IS) derivatives of chlorins and porphyrins. According to our IL and IS research the ability to finely adjust the biological properties of ionic liquids and surfactants by changing their anion/cation combination is a real benefit. ILs and Ionic surfactants are entirely appropriate and applicable to the field of photosensitizers and preparation of nanoparticles for screening of cancer cell, Photothermal therapy (PTT) and PDT of cancer.

## 3. Experimental Section

### 3.1. Synthesis of cholinium-purpurin-18

The Purpurin-18, cholinium hydroxide (CholOH) and Cholinium-Purpurin-18 (Chol-Pu-18) in this study were prepared using the general method for ionic liquid synthesis [9] and the method of preparation of Purpurin-18 derivitives [12].

Choline hydroxyde (0.001 mol) was dissolved in distilled water (100 mL) by heating and stirring. Purpurin-18 (0.001 mol) was dissolved in the solution of cholinium hydroxide (100 mL) by heating and stirring. The reaction mixture was heated and stirred for 2 h. Afterwards, the reaction mixture was cooled to room temperature, dichloromethane (100 mL) was added and the mixture was stirred for an additional 30 min. The two phases were separated and the solvent was removed from the dichloromethane phase on a rotary evaporator and the product was crystallized several times from cold acetone or acetonitrile to give the Chol-Pu-18-carboxylate (Chol-Pu-18) in 83 % yield.

The raw materaials and product were characterized by MS and NMR spectroscopy. Chol(OH):MS m/z 104 (M^+^), ^1^H NMR(500MHz, D_2_O) *δ* ppm 4.05(m,-O-CH_2_), 3.50(t,-CH_2_-),3.19 (s, 3CH_3_) and Pu-18: MS m/z 506(M^+^), ^1^H NMR(500MHz, CDCl_3_) *δ*, ppm 9.63(s,5-H), 9.38(s,10-H), 8.58 (s, 20-H), 7.90 (dd, *J* 18,11.7, 3a-H), 6.28 (d, *J* 11.5, 3b-H), 6.18 (d, *J* 9.1, 2b'H), 5.19 (d, 7-H), 4.39 (q, *J* 7.1, 18-H), 3.80,3.35 (each s, 3H and 12-CH_3_),3.63(q, *J* 7.5, 2H,8-CH_2_-CH_3_) 2.80 (m, 7b-H), 2.50 (m, 7b'H), 2.43 (m, 7a-H), 1.99 (m, 7a'H),1.75(d,8 *J* 7.1, Me),1.67(t, *J* 7.5, 4b-Me) and 1.25 and 0.93 (each b s, two NH,. s, NH).

The ^1^H NMR spectra of Chol-Pu-18 contains in addition to a Purpurin-18 structure an additional the multiplets, δ, ppm, 3.83(-O-CH_2_) triplets δ, ppm, 3.29(-CH_2_-) and singlet δ, ppm, 2.96 (-CH_3_) of the methylene and methyl groups decisively confirmed the structure of the quaternary ammonium ionic liquid salt.

### 3.2. Anticancer activity

The cells were illuminated with a LED light source UFPh-630/675-01-BIOSPEC (630–675 nm). The cancer cell samples were illuminated at a distance of 20 cm for 15 min, with light intensity of 40 mW/cm^2^ followed 3, 24, and 48 h later by optical microscopy to determine the morphological changes.

The each of cell lines were inoculated into a 96 wells, flat-bottomed microplate at a volume of 100μL for stationary culture. An amount of 2.5, 5.0, 10.0 and 20.0 μM were added to a volume of 100μL/well. Measurements were performed 3, 24 and 48 h after the irradiation.

### 3.3. Synthesis of gold nanoparticles

The gold nanoparticles were synthesized by Chol-Pu-18 self reduction of HAuCl_4_. The nanoparticles were synthesized according to the seed growth method [4,5] with some modifications.

*Preparation of seed solution*: 0.002 M Chol-Pu-18 solution (5 mL) was mixed with 0.001 M HAuCl_4_ (2.5 mL) in a 50 mL flat bottom flask and stirred in room temperature. This resulted in the formation of a black-green colored solution. After stirring for 2 hours, the solution was stored at room temperature.

*Growth of nanoparticles*: 0.001 M HAuCl_4_ (25 mL) is added to 0.002 M Chol-Pu-18 (25 mL) in a 250 mL flat-bottom flask and the solution changed color from yellow to green as the HAuCl_4_ dissolved in Chol-Pu-18. Then 0.005 M AgNO_3_ solution (1 mL) is added and mixed. To this solution seed solution (100 μL) was added to the center of the solution. After adding seed solution, the flask should be kept still and never moved, so that the seed started to grow in the growth solution. Usually the color of the solution gradually changed within few minutes. After few minutes, the gold nanoparticles are obtained and washed several time by water. In order to rule out the formation of nanoparticle aggregates on adsorption of the ionic liquid type of photosensitizers Chol-Pu-18, the coated gold nanoparticles were characterized by using Transmission Electron Microscopy (TEM). The Au nanoparticles were placed onto a copper grid with a small amount of water for TEM observation.
